# Supplementary material for: Benchmarking the predictive capability of human gait simulations
Source: PLoS Comput Biol. 2025 Nov 17;21(11):e1012713. doi: 10.1371/journal.pcbi.1012713 (PMC12622833; doi:10.1371/journal.pcbi.1012713)
Supplement: S1 File — (PDF) [file pcbi.1012713.s001.pdf]

Supplementary files

Methods

Table A:weights in the objective function

	weight	Unit
Metabolic rate squared	500	W^{-2}
Accelerations generalized coordinates squared	0.17	$rad^4 s^{-2}$ *and* $s^4 m^{-2}$
Muscle activations squared	2000	-
Passive joint moments squared	1000	$N^{-2}m^{-2}$
Torque actuators arms	10^6	$N^{-2}m^{-2}$
Slack control time derivative of muscle activation	10^-7	-
Slack control time derivative of normalized tendon force	10^-3	-

Results

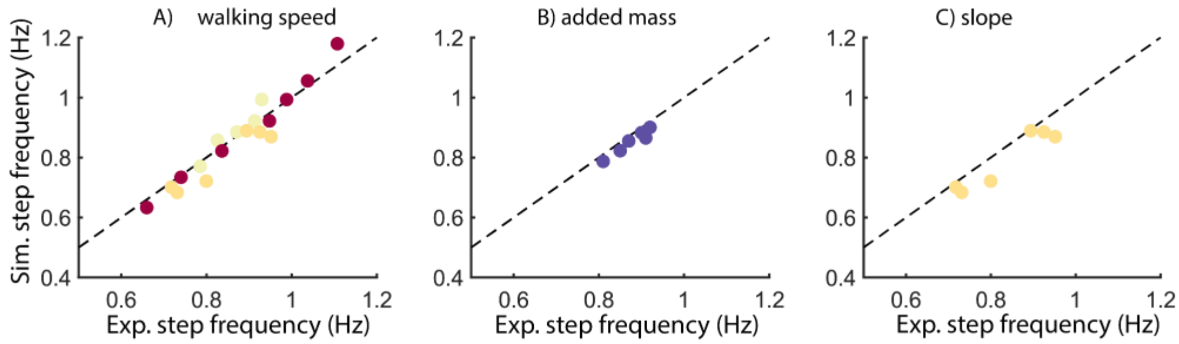

Figure A: absolute values of stride frequency for different interventions

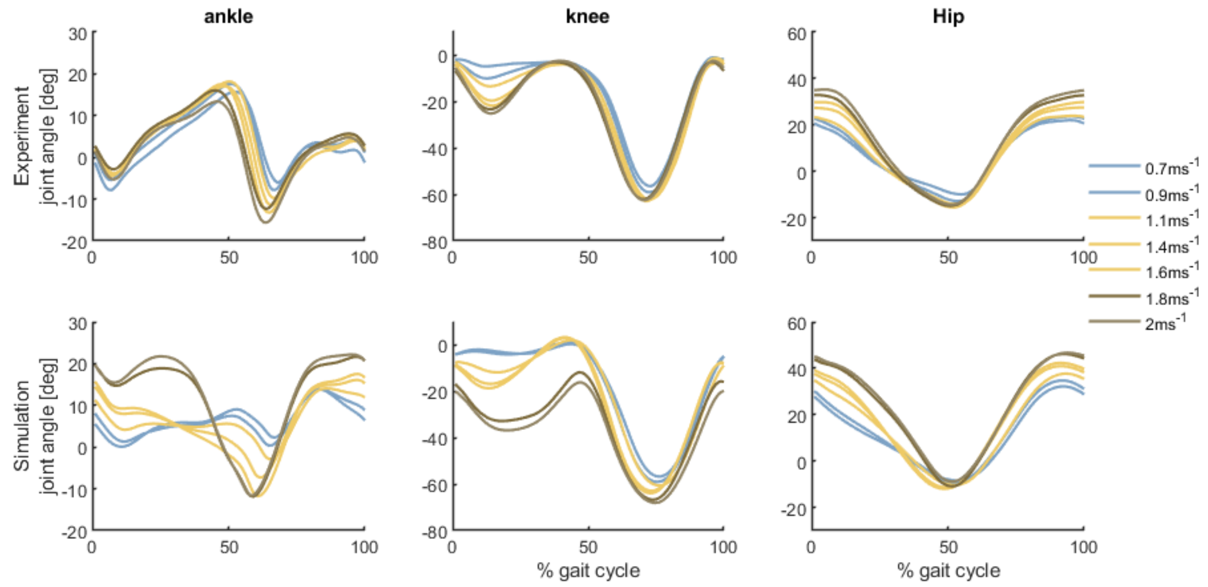

Figure B: Predicted and measured (Van Der Zee 2022) joint kinematics for various walking speeds (0.7, 0.9, 1.1, 1.4, 1.6, 1.8, 2 m/s). Experimental data was processed with AddBiomechanics.

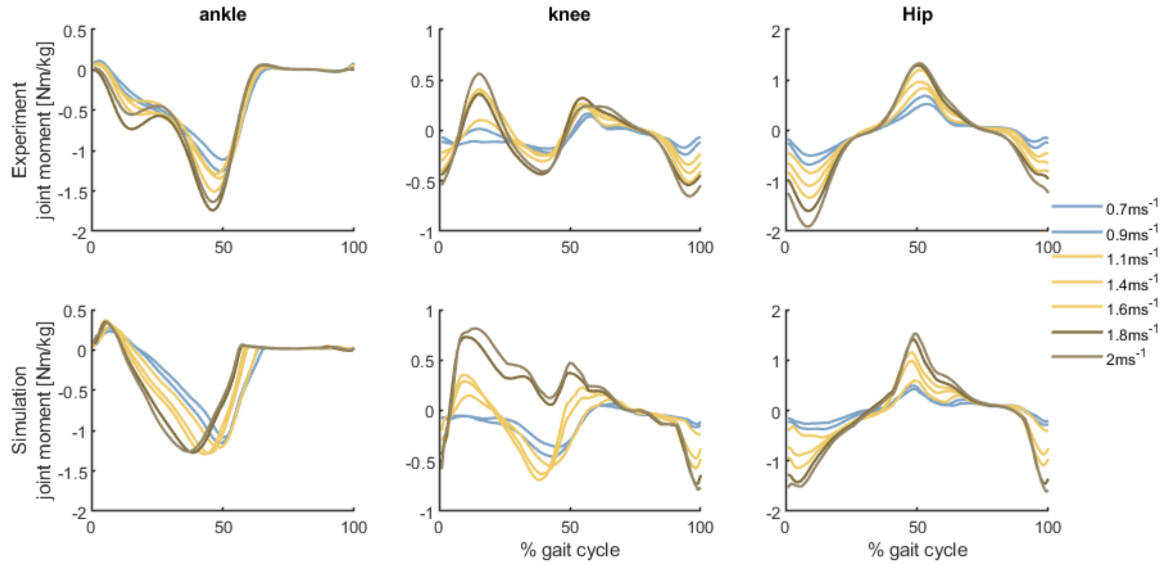

Figure C: Predicted and measured (Van Der Zee 2022) joint moments for various walking speeds (0.7, 0.9, 1.1, 1.4, 1.6, 1.8, 2 m/s). Experimental data processed was with addBiomechanics.

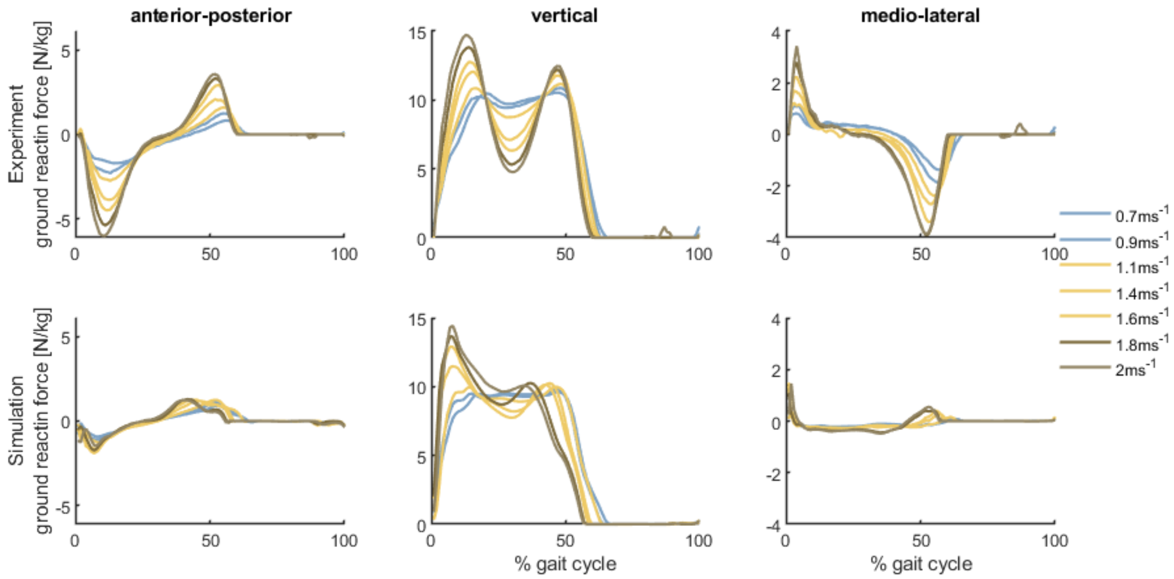

Figure D: Predicted and measured (Van Der Zee 2022) ground reaction forces for various walking speeds (0.7, 0.9, 1.1, 1.4, 1.6, 1.8, 2 m/s). Experimental data was processed with AddBiomechanics.

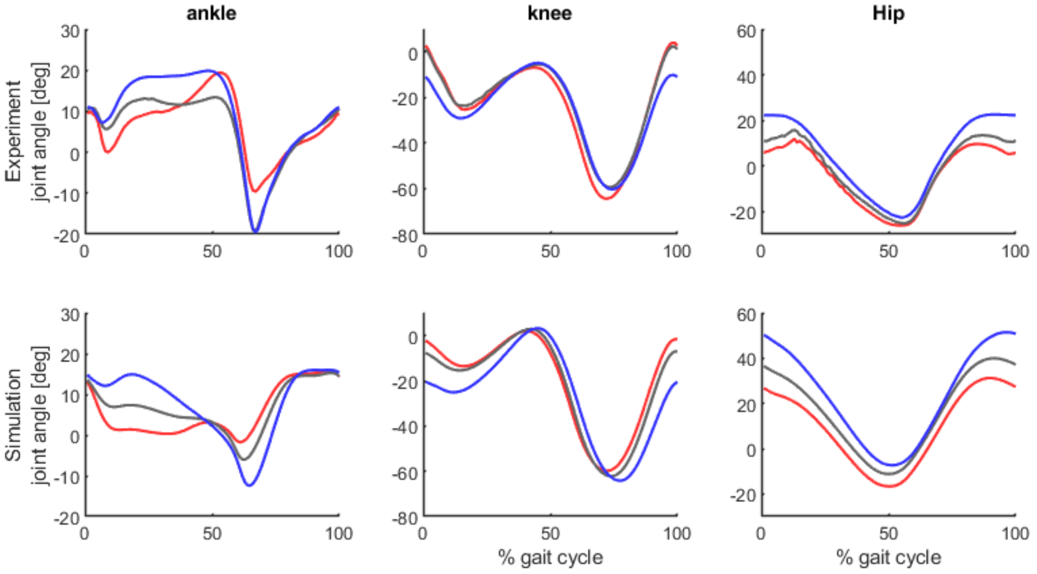

Figure E: Predicted and measured (Koelewijn 2019) kinematics for 8% downhill (red), level (level), and 8% uphill (blue) walking. Experimental data was processed with AddBiomechanics.

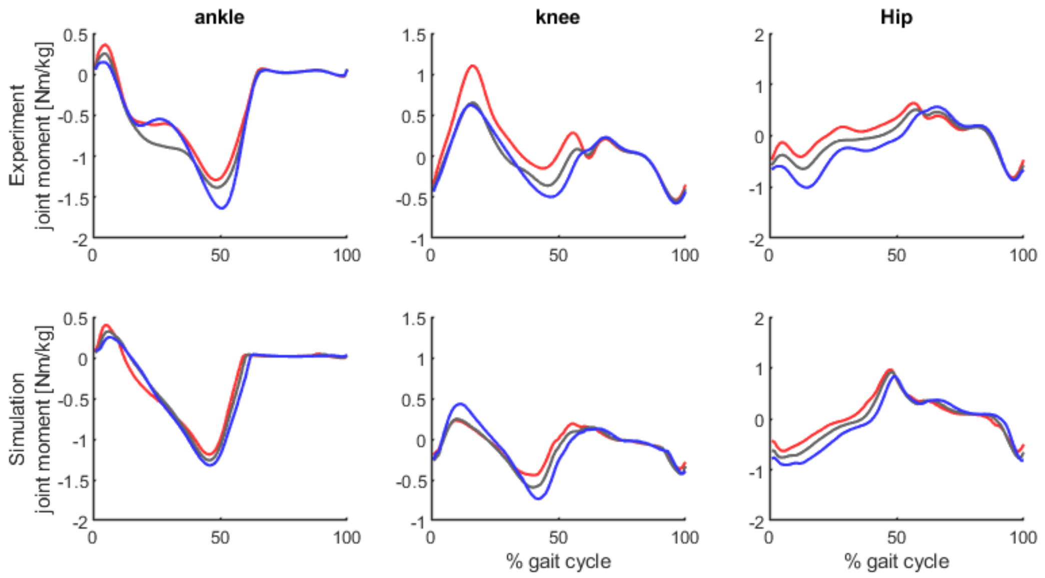

Figure F: predicted and measured (Koelewijn 2019) joint moments for 8% downhill (red), level (level), and 8% uphill (blue) walking. Experimental data was processed with AddBiomechanics.

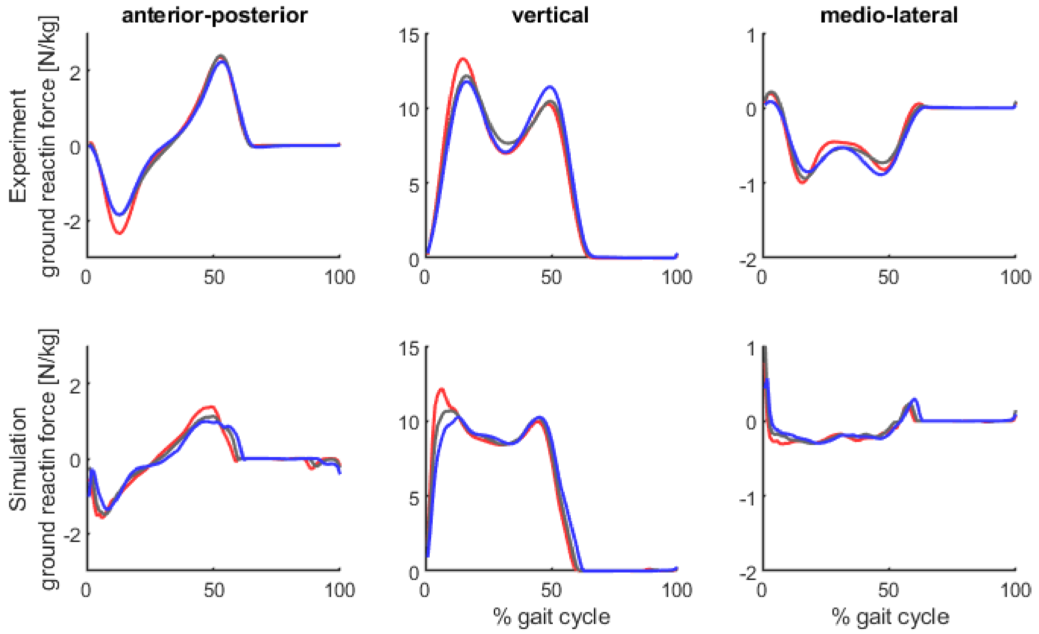

Figure G: predicted and measured (Koelewijn 2019) ground reaction forces for 8% downhill (red), level (level), and 8% uphill (blue) walking. Experimental data was processed with AddBiomechanics.

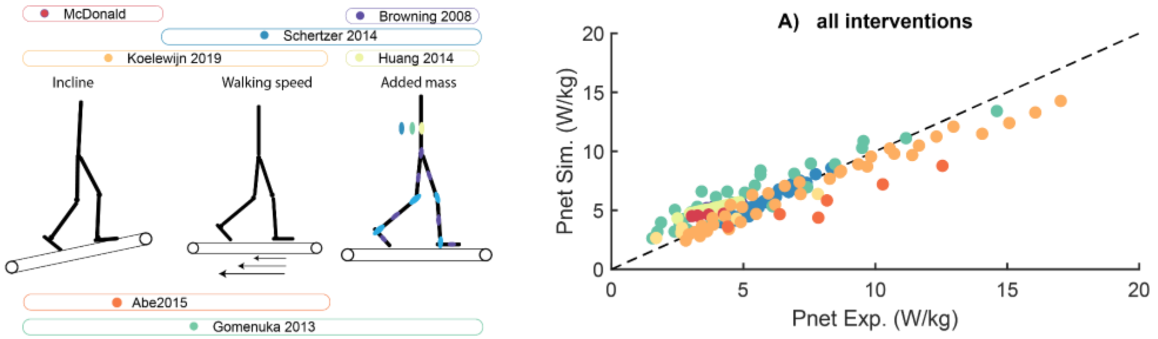

Figure H: Measured and predicted net metabolic power for different walking conditions including variations in walking speed, walking with added mass, and walking on a slope. Each dot represents the mean data reported in the different studies.

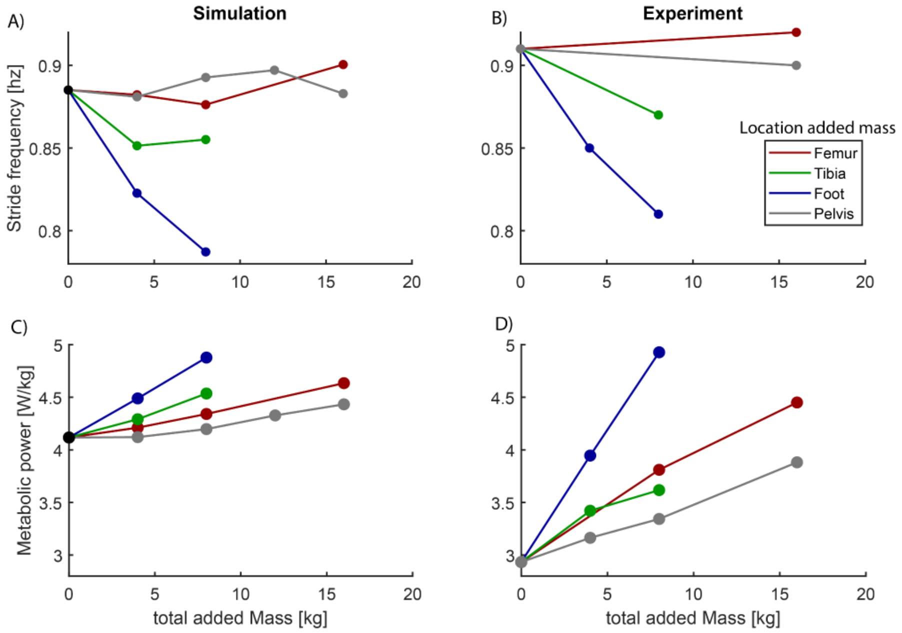

Figure I: Effect of location of added mass, i.e. pelvis, foot, tibia or femur, on simulated and measured stride frequency and metabolic power (experimental data from Browning 2008).

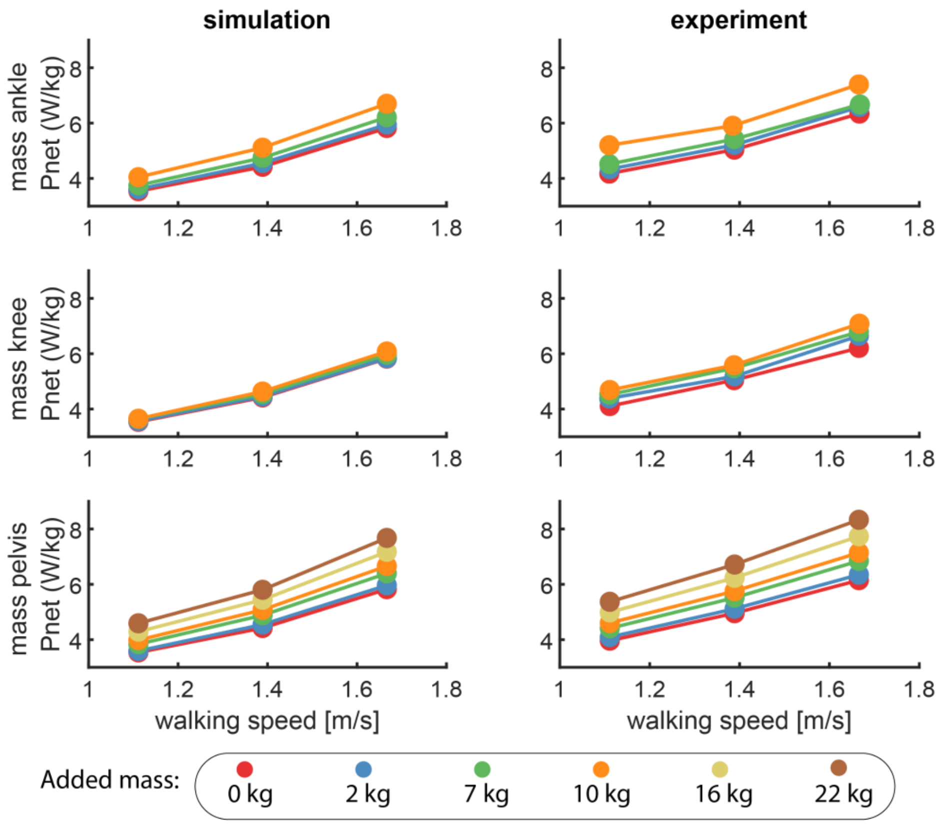

Figure J: Effect of location and magnitude of added mass on simulated and measured metabolic power (experimental data from Schertzer 2014).

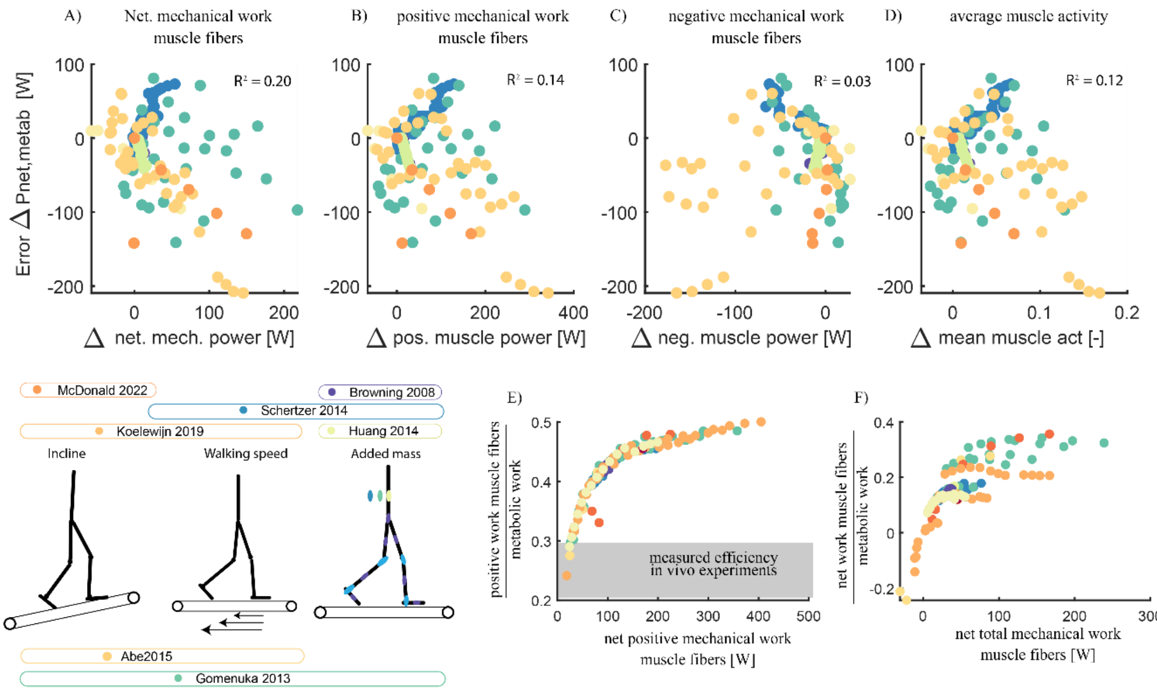

Figure K: We found that the underestimation of metabolic power was slightly related to the simulated net mechanical work during the task (A, R^2 = 0.2) and positive mechanical work (B, R^2 = 0.14) and average muscle activity (D, R^2= 0.12). There was no relation with negative mechanical work (C, Rsq = 0.03).

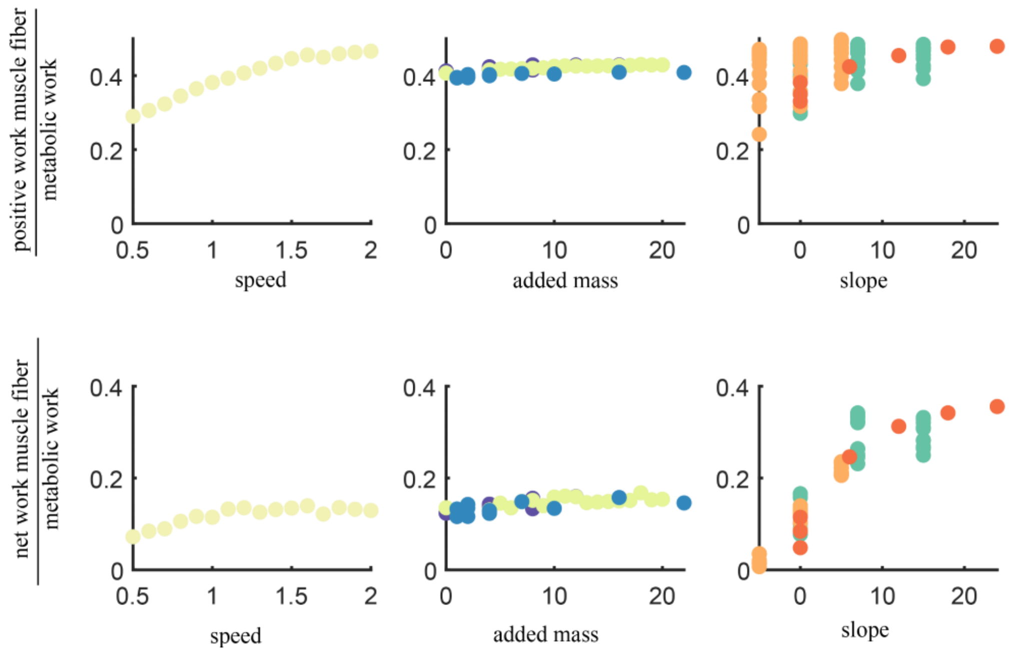

Figure L: Efficiency positive and net muscle fiber work for the different gait conditions.

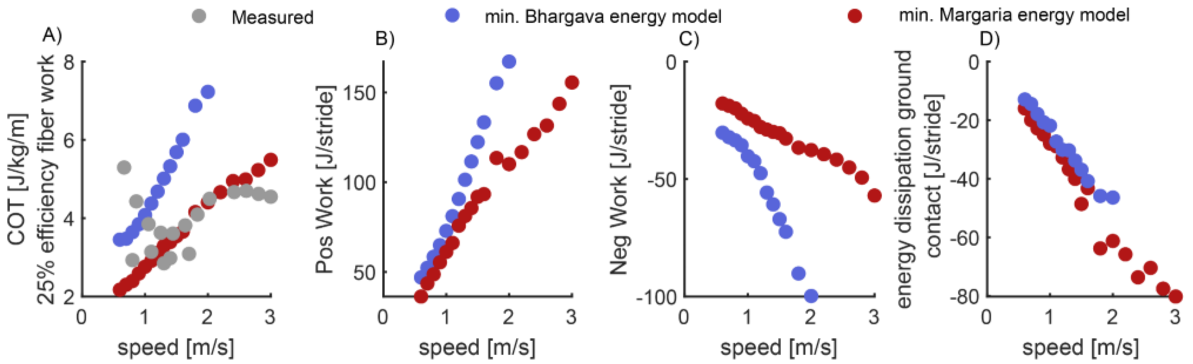

Figure M: Mechanical energy in simulations with Bhargava energy model (blue) or Margaria energy model (red) in cost function.

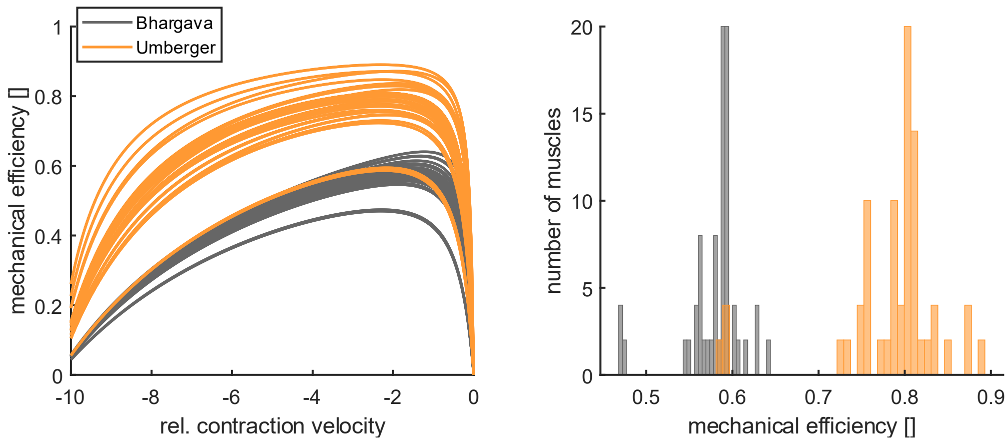

Figure N: maximal mechanical efficiency of the Bhargava and Umberger models of muscle energy expenditure.

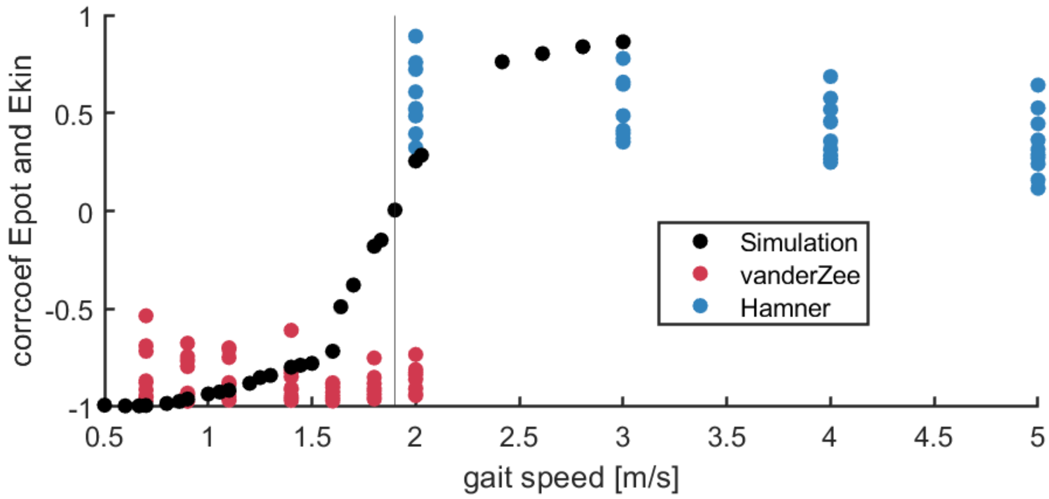

Figure O: correlation coefficient of the approximated kinetic and potential energy as a function of gait speed in the simulation (black), experiment van der Zee et al. where subjects were instructed to walk (red) and experiment of Hamner et al. where subjects were instructed to run (blue). The kinematics and potential energy where approximated based on the assumption that the skeletal system is a point mass located at the origin of the pelvis. A positive correlation coefficient can be interpreted as (grounded)running and a negative correlation coefficient as walking.

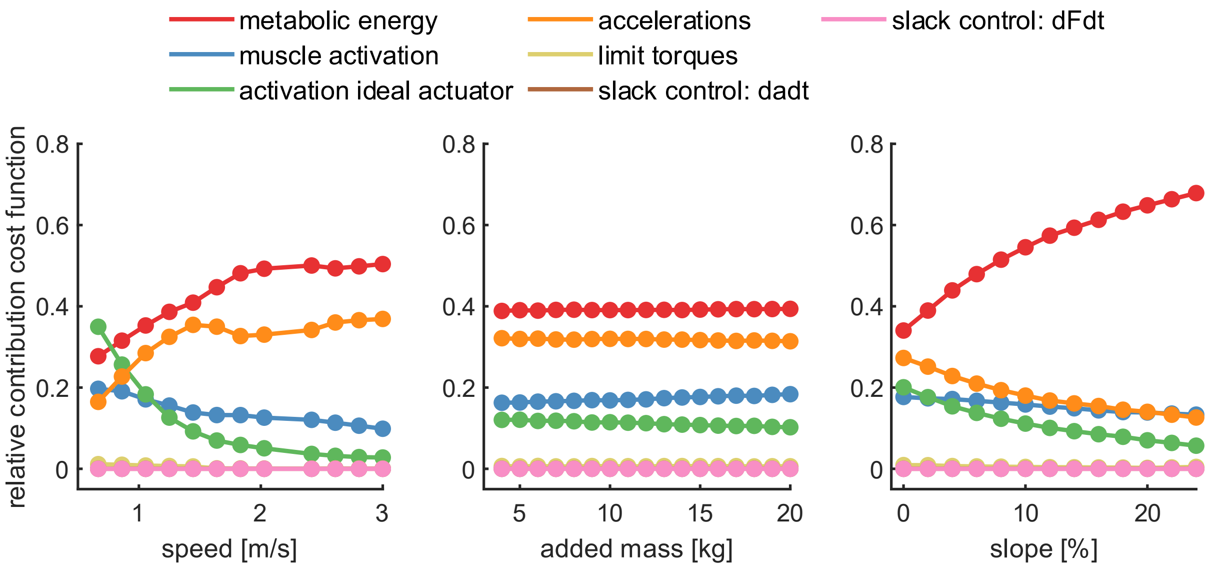

Figure P:Relative contribution of the different cost function components to the total cost function for level walking at various speeds, walking with at 1.25 m/s with added mass to the pelvis and walking at 1 m/s on various inclines.

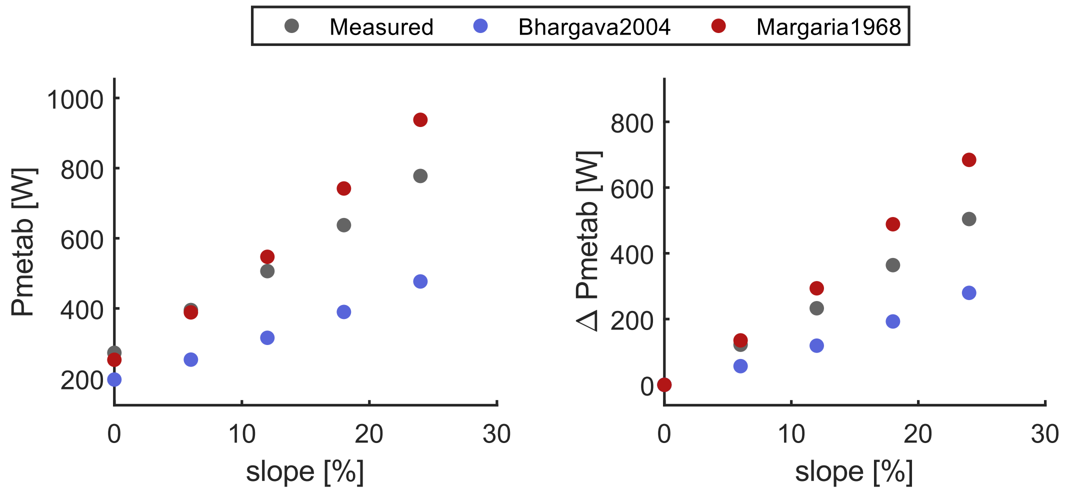

Figure Q: Absolute and relative increase average metabolic power for walking at 1m/s on various slopes in the experiments of McDonald et al.(gray), simulations processed with the Bhargava energy model (blue) and the Margaria energy model (red).
