# Supplementary material for: Benchmarking the predictive capability of human gait simulations
Source: PLoS Comput Biol. 2025 Nov 17;21(11):e1012713. doi: 10.1371/journal.pcbi.1012713 (PMC12622833; doi:10.1371/journal.pcbi.1012713)
Supplement: Attachment — Submitted filename: Review1.pdf [file pcbi.1012713.s003.pdf]

This paper describes a study in which predictive simulations are used to replicate several different experimental gait studies, with slope walking, added mass to the limbs, different walking speeds, among others. The goal is to investigate if different spatiotemporal, kinetic, kinematic, and energetic gait changes that were observed in the experiment can be predicted in the simulations. It was found that muscle variables were not estimated corrected: metabolic power was overestimated, which was likely related to the mechanical efficiency of muscles being overestimated, and so was the amount of positive work in the muscles, highlighting the need for more accurate muscle mechanics and energetics model.

Overall, this is great work, and it must have taken a lot of effort to complete. The paper is interesting to read and addresses and important goal: developing a benchmark for predictive simulations. However, the large number of experiments that were included (which is great) makes it difficult to follow the paper at times, due to the sheer amount of information. Furthermore, some critical information is missing in the manuscript due to the condensed presentation. Instead of increasing the length of the manuscript, which might reduce the readability, a supplement with detailed methods could be considered.

Major comments

- The authors call the model a "neuro-musculoskeletal model". Even though they do clearly describe what they mean with that, I still think that this term might put readers on the wrong foot. I agree that a neural model is included in the current work through the objective minimization, but it is not common to describe this as neuro-musculoskeletal model in the current literature. A neuro-musculoskeletal model usually implies that a neural control model is included, i.e., a set of mathematical equations describing the neural control as a function of the sensory inputs (e.g. a reflex model). Furthermore, because there is the musculoskeletal model and the objective, there are really two separate model types, and "neuro-musculoskeletal model" implies that it is a single thing.
- Line 38, 216. The authors claim that the implemented tasks are straightforward, but there are some obvious differences between how the tasks were measured experimentally and how they were implemented. Most notable, there is a difference between treadmill walking and overground walking. Therefore, I would recommend wording changes throughout the manuscript and clarifying these differences throughout the manuscript.
    - Line 38: "ensuring" seems a bit too strong here.
    - Line 94: "ensuring that prediction errors …" is also too strong.
    - Line 216: a simulation with a forward pelvis speed is actually different from a treadmill experiment.
    - The discussion should include more reflection on the difference between experiment and simulation. Would a treadmill model improve the accuracy of the simulations? Are there any other differences? The reflection could

also include more reflection on the metabolic power over- and underestimation (line 362-364) and on why some of the experiments were implemented differently in the simulation than in the experiment (line 223 and 336)

- Line 101: I would not say that the objective "typically comprises" all those terms listed in lines 100-102, based on the literature. Please rewrite. While the objective has been used in at least 2-3 papers from the authors' group, there are many more predictive simulation studies that use other objectives, such as:
    - Lin, Y. C., Walter, J. P., & Pandy, M. G. (2018). Predictive simulations of neuromuscular coordination and joint-contact loading in human gait. Annals of biomedical engineering, 46, 1216-1227.
    - Ackermann, M., & Van den Bogert, A. J. (2010). Optimality principles for model-based prediction of human gait. Journal of biomechanics, 43(6), 1055-1060.
    - Miller, R. H., & Esposito, E. R. (2021). Transtibial limb loss does not increase metabolic cost in three-dimensional computer simulations of human walking. PeerJ, 9, e11960.
    - Miller, R. H., & Hamill, J. (2015). Optimal footfall patterns for cost minimization in running. Journal of Biomechanics, 48(11), 2858-2864.
    - Koelewijn, A. D., & Selinger, J. C. (2022). Predictive simulations to replicate human gait adaptations and energetics with exoskeletons. IEEE Transactions on Neural Systems and Rehabilitation Engineering, 30, 1931-1940.
    - Dorschky, E., Krüger, D., Kurfess, N., Schlarb, H., Wartzack, S., Eskofier, B. M., & van den Bogert, A. J. (2019). Optimal control simulation predicts effects of midsole materials on energy cost of running. Computer methods in biomechanics and biomedical engineering, 22(8), 869-879.
    - Ong, C. F., Geijtenbeek, T., Hicks, J. L., & Delp, S. L. (2019). Predicting gait adaptations due to ankle plantarflexor muscle weakness and contracture using physics-based musculoskeletal simulations. PLoS computational biology, 15(10), e1006993.
- Relatedly, I think that the introduction and discussion could explore the current literature on predictive simulations more. The authors made specific (and logical) choices for the objective and modelling of the musculoskeletal model, but for both, there are other options. For the objective, it is as described above, while other studies in the list above have included body variations through virtual participants in different ways. It would be good to describe these options in more detail in the introduction and also reflect on those in the discussion. For example, I would expect that the results in, e.g., Fig 3, are highly dependent on the choice of walking objective.
- Line 191: normally, the initial state of the simulation is also identified through the optimal control problem. Please add that to the description

- Line 279: I am a bit confused by the description, because I interpreted the description as though the measured metabolic energy was used to calculate the efficiency and not the estimated net energy from the simulation? Or am I missing something?
- Line 300: what is meant with, e.g., 10% overestimation and how was it calculated?
- Results (e.g., Figure 2): In general, I am not sure if a simple correlation is valid for the comparisons that are made here. A simple correlation assumes independent data, but this is violated by the fact that there are multiple experiments. In fact, you can see an effect of the experiment in the graphs. I am not an expert myself, but I would think that something like a linear mixed model would be required to adjust for the experiment factor.
- Figure 3: I did not fully understand the analysis that was done here and would need to dive back into the original experimental paper. Therefore, please explain figure D-G more, so that they can be interpreted without reading the original paper.
- Figure 4: Please explain Figured C-F more. It is not clear how these results are generated, specifically the different lines and the "number of muscles".
- Figure 6: Figure C is not referred to in the manuscript. Consider removing or adding a reference.
- Line 558: How is the COT with reduced dissipation larger than without? I compared to the previously mentioned value, 5.03 in line 543. Please clarify.
- Line 600-601: the comparison that is made here is unfair. On the one hand, the simulation probably assumes an idealized exoskeleton with any possible control that spans all joints in the legs, while such an exoskeleton cannot be created in practice, and there are limitations in the connection between exoskeleton and person.

Minor comments

- Line 37: inclined walking
- Line 53: interaction instead of interacting
- Line 62: control system and line 68: metabolic power model are maybe a bit too technical for the author summary
- Line 116: consider adding the following reference: Jessup, L. N., Kelly, L. A., Cresswell, A. G., & Lichtwark, G. A. (2023). Validation of a musculoskeletal model for simulating muscle mechanics and energetics during diverse human hopping tasks. *Royal Society Open Science*, *10*(10), 230393.
- Line 173: hip, knee, ankle, shoulder, elbow should be plural
- Line 200: add subscript to w1-w5 to match the style in equation 1.
- Line 202: MATLAB
- Line 241 addBiomechanics

- Table 1: consider using participants instead of subjects (see https://pubmed.ncbi.nlm.nih.gov/38075407/)
- Line 273: qualitatively should come at the end of the sentence
- Line 504: capitalize Hill
- Line 511: experiments (plural)
- Line 522: add a citation to “recent simulation work”
- Line 536: This paragraph only discusses inaccuracies in the muscle dynamics. Consider rephrasing
- Line 587: this paragraph is not well connected to the rest of the manuscript.
- Line 631: we instead of I?
- Ref 20: authors are missing.
