# Supplementary material for: Benchmarking the predictive capability of human gait simulations
Source: PLoS Comput Biol. 2025 Nov 17;21(11):e1012713. doi: 10.1371/journal.pcbi.1012713 (PMC12622833; doi:10.1371/journal.pcbi.1012713)
Supplement: Attachment — Submitted filename: Response reviewers.docx [file pcbi.1012713.s005.docx]

We want to thank both reviewers for their detailed review of the research article and insightful questions. You can find your questions in blue in this document and my answers in black and text copied from the new version of the manuscript in italic.

***Reviewer 1:***

**General comments**

Line 156-157. Cost function weights were hand-tuned to obtain good predictions for a single walking condition. This is somewhat contradictory to what was said in the previous paragraph, that such an approach can lead to overfitting, and produce poor predictions for cases outside of the “training” data. While I agree with this approach, scientifically (you benchmark an existing method), it’s good to keep in mind that the cost function may not have been the best one. This can be addressed in the Discussion. See a later comment.

We agree with the reviewer. Since the cost function was hand-tuned to yield good predictions for a single walking condition, we primarily evaluated its ability to predict changes in kinematics, kinetics, and energetics relative to this condition (i.e., outside the training data). We also agree that the influence of the cost function on the predicted gait patterns should be discussed, and we have now added this point to the Discussion section.

Adapted paragraph in discussion section:

*The optimality assumption and the particular cost function used might also contribute to errors in gait mechanics and metabolic power. Both the terms and their relative weights in the cost function shape the predicted gait pattern and thus the predicted metabolic power* [1]*. It is unlikely that the objective function used here is an accurate representation of the gait-related cost minimized by humans for two reasons. First, the terms and weights in the cost function were determined based on a trial-and-error approach based on the realism of the simulated walking pattern at 1.3 m/s. It is unlikely that experimental data of walking at 1.3 m/s contains enough information to identify the cost function resulting in a considerable risk of overfitting. Instead, the cost function should be identified from a comprehensive dataset spanning a range of gait conditions that allow dissociating the different cost function terms. We found that the relative contribution of different terms (e.g., muscle activations vs. metabolic energy) varied across our simulated gait tasks (Figure S16). Identification of the cost function is further complicated by the correlations between different candidate cost function terms. Yet, progress is to be expected from using data of carefully designed experiments* [2]*. Second, identification of the cost function is complicated by modeling errors. The specific cost function terms and weights might be required to obtain realistic gait patterns in the presence of errors in the musculoskeletal or energy models. For example, there is no experimental evidence for squaring the metabolic rate or adding joint accelerations. Maybe such terms might not be needed with better metabolic cost models or when accounting for uncertainty (e.g. due to sensorimotor noise). It is most likely a good idea to replace activation terms by volume-scaled activation terms as the activation cost is sensitive to splitting muscles in different bundles whereas volume-scaled activation is not* [3]*. Finally, inaccuracies in the cost function, or in the neural control model in general, may also affect how co-contraction is modeled, which in turn influences simulated mechanical work* [4]*. Increased co-contraction increases energy dissipation in antagonistic muscles, which has to be compensated by increased positive work in agonistic muscles. It is however unlikely that high levels of co-contraction cause the large amount of positive and negative mechanical work in our simulation as co-contraction was implicitly minimized in our cost function (activation squared and metabolic energy term).*

Line 159. The term “linear muscle actuators” is not familiar to me. Please reword or clarify with a reference.

This is indeed a bit short. We wanted to point out that muscle paths were described by line segments in our model. We removed this example because it was not further discussed(end introduction section).

Equation 1: The notation is potentially confusing to someone who is not familiar with previous work. You could either explicitly do summations over muscles, degrees of freedom, etc., or use the notation from Falisse et al. (2019) to show that each term is actually a squared vector norm over multiple components.

Agreed, this was a bit sloppy. We adapted the equation.

$$J=\frac{1}{d}\int_{0}^{t_{f}} \left( \underset{\text{Metabolic} \text{power}}{\underbrace{w_{1}\parallel\dot{E}\parallel_{2}^{2}}}+\underset{\text{Muscle} \text{activity}}{\underbrace{w_{2}\parallel a\parallel_{2}^{2}}}+\underset{\text{Joint} \text{acc}}{\underbrace{w_{3}\parallel\ddot{q}\parallel_{2}^{2}}}+\underset{\text{Passive} \text{torques}}{\underbrace{w_{4}\parallel T_{\text{p}}\parallel_{2}^{2}}}+\underset{\text{Arm} \text{excitations}}{\underbrace{w_{5}\parallel e_{\text{arms}}\parallel_{2}^{2}}}+\underset{\text{Slack} \text{controls}}{\underbrace{w_{6}\parallel u_{\text{slack}}\parallel_{2}^{2}}} \right)\text{d}t,$$

Line 200, “We used the weights (w1- w6) as published in (21).” The reference (21) does not provide values for the weights, perhaps (10) should be used as a reference. Also, it would be helpful to list the weights in a Table (in Supplement). When providing numerical values for the weights, it is important to specify the units of the variables they are applied to, i.e. energy rates must be in Watts, generalized accelerations in m/s^2 or rad/s^2, etc. Otherwise the weight values are not meaningful.

Agreed. The weight values are essential to replicate our results. We added the weight values to the supplementary file (Table S1) and refer to this table in the methods section.

*Table S1:weights in the objective function*

|  | **weight** | **Unit** |
| --- | --- | --- |
| Metabolic rate squared | 500 | $W^{-2}$ |
| Accelerations generalized coordinates squared | 0.17 | $rad^{4}s^{-2} and s^{4}m^{-2}$ |
| Muscle activations squared | 2000 | - |
| Passive joint moments squared | 1000 | $N^{-2}m^{-2}$ |
| Torque actuators arms | 10^6 | $N^{-2}m^{-2}$ |
| Slack control time derivative of muscle activation | 10^-7 | - |
| Slack control time derivative of normalized tendon force | 10^-3 | - |

Lines 330-331. “The observed increase in hip flexion moment and extension moment with faster walking was predicted accurately”. However, Figure S4 shows that the horizontal ground reaction force (in both horizontal directions) is severely underestimated. It seems unlikely that the hip moment is predicted correctly when the horizontal GRF is not. It may be that there are also prediction errors in vertical GRF and/or kinematics, in such a way that the total effect of these errors on hip moment is largely canceled out. The error in horizontal GRF should be mentioned and discussed, because it suggests that the model does not walk like the human participants. In fact, Figure S4 is never mentioned in the manuscript at all.

Agreed, this was added to the results section. This line now reads:

*The simulations also failed to capture the increase in magnitude of the anterior-posterior component of the ground reaction force with increased gait speed (Figure S4).*

Lines 396-397. “The simulation did not account for the total activated muscle volume, which could have influenced the results (Figure 3 G).” Please clarify what is meant by “did not account for”.

We have revised the manuscript accordingly: the explanation was removed from the figure caption and integrated into the Results and Discussion sections. By “did not account for,” we meant that the objective function minimized muscle activations squared rather than volume-weighted muscle activations squared.

Novel part in the results section:

*The metabolic power component of the cost function was lower for crouched walking than for slope walking at all tested slopes above 0% whereas experimentally metabolic power of crouch walking is similar to walking with a slope of 12% incline (Figure 3E). Interestingly, we also observed that the muscle activation component in the cost function for crouch walking was lower than for all tested slopes; the relative activation cost of crouch versus slope walking is thus different for the subset of muscles that were experimentally assessed and all the muscles (Figure 3F). The activation cost of a subset of muscles might thus not be representative for all muscles. The muscle activation component in our cost function was not dependent on muscle volume (i.e., the activation of muscles with small and large volumes contributed equally in the cost function). When we postprocessed the results using relative muscle volumes from* [2]*, we found that this weighting altered the activation component in the cost function for both crouch and slope walking (Figure 3G).*

Figure 3G. The Figure says “muscle mass”, while the text says “muscle volume”, so it is not clear what it actually is. It would help to explain how this variable is calculated, and place the appropriate unit (kg or m^3) on the vertical axis.

It should have been relative muscle volumes. We computed volume-weighted muscle activations during postprocessing of the simulation results to replicate the outcomes reported by McDonald et al. [2]. I therefore used the values provided by McDonald for the relative weights of the muscles in the model. To paraphrase the paper: “These weightings were 0.33, 0.08, 0.10, 0.17, 0.10, 0.17and 0.05 for Gmax, BF, RF, VM, MG, SOL and TA, respectively, and represent each muscle’s volume as a proportion of the sum of all seven muscles”.

This was adapted in the results section (see answer the previous question in this document).

This also reminds me of an important paper by Holmberg and Klarbring (2012) which proved that muscle activations (or squared activations) should not be added up in an optimization objective without weighting them by factors that make the objective invariant to anatomical discretization. Muscle volume, or mass, or PCSA, would all be appropriate weighting factors. Sometimes, in 3D models, a muscle is divided into many components, each with their own line of action. Such muscles then get too much weight in an activation-based optimization objective, unless their contributions are weighted appropriately. This may be good to mention in the Discussion, in the paragraph about the optimization objective.

We completely agree with the reviewer. The example of discretizing muscles into multiple lines of action illustrates very clearly why our current implementation is flawed. The only reason we did not adjust this is that our goal was to benchmark an existing method without suggesting that we fine-tuned it to fit the benchmark test data. We have added this point to the Discussion section. We also thank the reviewer for pointing us to the reference by Holmberg and Klarbring (2012), which is indeed a valuable reference in our discussion.

Adapted discussion section:

*It is most likely a good idea to replace activation terms by volume-scaled activation terms as the activation cost is sensitive to splitting muscles in different bundles whereas volume-scaled activation is not [3].*

Line 492. “The underestimation of metabolic power”. In fact, the model overestimated the metabolic power! It is only the change in metabolic power that is underestimated. Please make this distinction. It is not clear to me how an unrealistically high muscle efficiency can cause the metabolic power to be overestimated. Nevertheless, the changes were underestimated. Could it be that the metabolic power had too much weight in the optimization objective, causing gait adaptations to avoid increasing it?

We have adapted the manuscript to clarify that our statement referred to the change in metabolic power relative to level walking. As noted, depending on the gait condition, the absolute metabolic power was either over- or underestimated (Figure S8). However, for walking on a slope, the metabolic power was always underestimated in both absolute and relative terms when using the Bhargava (and Umberger) energy models, consistent with the findings of Koelewijn et al. [3]. Since walking on a steep incline requires net positive muscle fiber work, this suggests that the model’s treatment of mechanical efficiency is problematic.

Regarding the reviewer’s question: at first glance we were also surprised that an unrealistically high efficiency can still lead to overestimations of metabolic power in conditions such as fast walking. The fact that a model that is too efficient overestimates metabolic power points to other fundamental modeling errors. To explore this further, we post-processed our simulation results with an energy model that assumes a more plausible mechanical efficiency of positive and negative muscle work (Margaria model). This analysis revealed even larger overestimations of metabolic power for fast walking, in both relative (Figure 5) and absolute terms (Figure 6A). These results show that the main issue lies in a large overestimation of positive and negative mechanical work by the muscle fibers in the simulations. This effect was particularly evident in fast walking, where the net mechanical work is small (mainly to compensate for energy lost during ground contact and joint damping) and the positive and negative mechanical work done by muscle fibers is large.

As for the reviewer’s suggestion: indeed, the weight assigned to metabolic energy in the optimization objective could influence the amount of positive and negative muscle fiber work in the simulations. A higher weight might reduce fiber work, thereby changing the predicted metabolic power. We also observed that gait patterns differed substantially when the Margaria energy model was used in the objective function compared to the Bhargava model. So it seems that with our current model it is impossible to predict both realistic walking kinematics and accurate predictions of positive and negative muscle fiber work.

Your question makes it clear that our reasoning/flow of thoughts was not clear in the original manuscript. As this is a major point in our paper, we added a new paragraph to the discussion section and adapted several parts of the discussion section slightly to account for this remark.

New paragraph in discussion section:

*It seems unlikely that changing the cost function could yield both realistic walking kinematics and accurate predictions of positive and negative muscle fiber work in our simulations. Minimizing metabolic energy with the Margaria energy model (based on efficiencies of positive and negative fiber work) produced realistic metabolic costs for walking at different speeds (Figure 6A, red and gray dots), but at the expense of poor kinematic and kinetic predictions (Figure 6C–I). The Margaria model likely underestimates the metabolic energy due to not accounting for the cost of isometric contractions, and therefore our simulations based on minimizing metabolic energy according to Margaria indicate that errors in musculoskeletal dynamics rather than only the cost function cause unrealistically high muscle work. Similar trends are evident in other simulation studies that applied the Margaria model to estimate the metabolic cost of walking based on experimental data of walking kinematics. For example, Koelewijn et al.* [5] *also reported overestimations of metabolic power during level walking, consistent with an overestimation of positive and negative fiber work. Moreover, they also found that the Bhargava* [6] *and Umberger* [7], [8] *models yielded realistic metabolic power during level walking, but substantially underestimated metabolic power for slope walking. Given that Koelewijn et al. used an inverse approach with a different cost function to solve the muscle redundancy problem, similarities between their and our findings further confirm that the apparent agreement between simulated and measured metabolic power during level walking (Figure 2) likely stems from overly high mechanical efficiency of the metabolic energy model masking the unrealistically high predicted muscle fiber work.*

Line 501. Similar to the previous comment, “underestimation of metabolic cost”, should be “underestimation of changes in metabolic cost”.

There is an underestimation of metabolic cost both in relative and absolute terms for uphill walking. Note that this was also reported in Koelewijn et al [5] (Bhargava and Umberger energy models). I added a figure to the appendix to make clear that for uphill walking there is always an underestimation of metabolic cost.

*Figure S17: Absolute and relative increase in average metabolic power for walking at 1m/s on various slopes in the experiments of McDonald et al.(gray), simulations processed with the Bhargava energy model (blue) and the Margaria energy model (red).*

Lines 578-580. These two sentences state an obvious but important fact, but then the topic is changed without any discussion of the optimization objective. This should be significantly expanded. The optimization objective is directly taken from Falisse et al. (2019). Different weights, or different objectives altogether, could be considered. In a previous comment, I already mentioned that the activation objective is not properly weighted. Furthermore, others (Ackermann 2010, Rasmussen 2001) have suggested fatigue-like criteria such as minimizing only the largest muscle activation (which then is no longer subject to Holmberg and Klarbring’s concerns). Also, the squaring of each muscle’s energy rate before adding and integrating certainly does not result in anything resembling the cost of transport. I know that Falisse tried without squaring and did not like the predictions, but physiologically, this objective makes no sense. Also, the cost function terms related to energy, activation, and generalized accelerations are probably highly correlated. It may well be that Falisse could have obtained equally good predictions by weighting them differently. In that case, there was overfitting, which could explain some of the prediction errors in this important benchmark study. I believe there is much work to do on cost functions, and testing them with benchmark tests exactly as presented in this manuscript. It is scientifically acceptable to use just one (previously published) cost function in this paper, but it is not acceptable to discuss the results without a robust discussion of possible problems with the cost function. The reader deserves more than just stating the obvious fact that the cost function influenced the predictions.

We fully agree with the reviewer’s comments. The choice of objective function is indeed an important aspect of human gait simulations, and in our study there are several limitations, including (1) not weighting activations by muscle volume, and (2) integrating squared metabolic power. Both are clearly not physiological and were adopted for practical reasons (e.g., numerical convergence issues when not squaring metabolic power). More fundamentally, there is a lack of experimental data to robustly inform the formulation of cost functions. For instance, studies such as McDonald et al. [2] provide insight into the relative importance of minimizing (volume-weighted) muscle activations versus metabolic power. Other researchers (e.g., [9]) have optimized cost function weights by minimizing differences between simulated and measured kinematics and kinetic, an approach that, as in our case with hand-tuned weights, risks overfitting if not validated against independent gait conditions.

We agree that the discussion deserves more than the statement that the cost function influences predictions. We have therefore expanded the Discussion section at several points to explicitly address the limitations of the chosen cost function, the potential impact of alternative formulations, and the need for further benchmark studies to systematically evaluate them.

Adapted paragraphs in the discussion section. Note these adaptations were already introduced in the previous questions.

*The optimality assumption and the particular cost function used might also contribute to errors in gait mechanics and metabolic power. Both the terms and their relative weights in the cost function shape the predicted gait pattern and thus the predicted metabolic power* [1]*. It is unlikely that the objective function used here is an accurate representation of the gait-related cost minimized by humans for two reasons. First, the terms and weights in the cost function were determined based on a trial-and-error approach based on the realism of the simulated walking pattern at 1.3 m/s. It is unlikely that experimental data of walking at 1.3 m/s contains enough information to identify the cost function resulting in a considerable risk of overfitting. Instead, the cost function should be identified from a comprehensive dataset spanning a range of gait conditions that allow dissociating the different cost function terms. We found that the relative contribution of different terms (e.g., muscle activations vs. metabolic energy) varied across our simulated gait tasks (Figure S16). Identification of the cost function is further complicated by the correlations between different candidate cost function terms. Yet, progress is to be expected from using data of carefully designed experiments* [2]*. Second, identification of the cost function is complicated by modeling errors. The specific cost function terms and weights might be required to obtain realistic gait patterns in the presence of errors in the musculoskeletal or energy models. For example, there is no experimental evidence for squaring the metabolic rate or adding joint accelerations. Maybe such terms might not be needed with better metabolic cost models or when accounting for uncertainty (e.g. due to sensorimotor noise). It is most likely a good idea to replace activation terms by volume-scaled activation terms as the activation cost is sensitive to splitting muscles in different bundles whereas volume-scaled activation is not* [3]*. Finally, inaccuracies in the cost function, or in the neural control model in general, may also affect how co-contraction is modeled, which in turn influences simulated mechanical work* [4]*. Increased co-contraction increases energy dissipation in antagonistic muscles, which has to be compensated by increased positive work in agonistic muscles. It is however unlikely that high levels of co-contraction cause the large amount of positive and negative mechanical work in our simulation as co-contraction was implicitly minimized in our cost function (activation squared and metabolic energy term).*

*It seems unlikely that changing the cost function could yield both realistic walking kinematics and accurate predictions of positive and negative muscle fiber work in our simulations. Minimizing metabolic energy with the Margaria energy model (based on efficiencies of positive and negative fiber work) produced realistic metabolic costs for walking at different speeds (Figure 6A, red and gray dots), but at the expense of poor kinematic and kinetic predictions (Figure 6C–I). The Margaria model likely underestimates the metabolic energy due to not accounting for the cost of isometric contractions, and therefore our simulations based on minimizing metabolic energy according to Margaria indicate that errors in musculoskeletal dynamics rather than only the cost function cause unrealistically high muscle work. Similar trends are evident in other simulation studies that applied the Margaria model to estimate the metabolic cost of walking based on experimental data of walking kinematics. For example, Koelewijn et al.* [5] *also reported overestimations of metabolic power during level walking, consistent with an overestimation of positive and negative fiber work. Moreover, they also found that the Bhargava* [6] *and Umberger* [7], [8] *models yielded realistic metabolic power during level walking, but substantially underestimated metabolic power for slope walking. Given that Koelewijn et al. used an inverse approach with a different cost function to solve the muscle redundancy problem, similarities between their and our findings further confirm that the apparent agreement between simulated and measured metabolic power during level walking (Figure 2) likely stems from overly high mechanical efficiency of the metabolic energy model masking the unrealistically high predicted muscle fiber work.*

To help explain some incorrect predictions, and give guidance for future research, it might be helpful to have some plots of the five (weighted) cost function components as a function of the test condition (speed, added weight, etc.). Then we might see if any component is dominating, and how each of them might be driving the gait adaptations. This could be in the Supplement and be cited in the discussion of the optimization objective.

Nice idea, I added this to the appendix (Figure S16) and refer to this in the manuscript.

*Figure S16:Relative contribution of the different cost function components to the total cost function for level walking at various speeds, walking with at 1.25 m/s with added mass to the pelvis and walking at 1 m/s on various inclines.*

Feeback on github repository

Many thanks for taking the effort to closely look at the code provided in the repository and sorry that the code did not run effortlessly. I did some major changes and tried to improve the code and the organization of all files.

In addition, I’m working on a simple implementation in the PredSim framework (see branch bechmarking: <https://github.com/KULeuvenNeuromechanics/PredSim/tree/benchmarking> ). If you would like to a look at the benchmarking functionality, please run this workflow.

**Minor changes in manuscript:**

Line 41. “kinematic” should possibly be “kinematics”.

I adapted this in the manuscript, this line now reads: “”.

*The simulations predicted stride frequency and walking kinematics with reasonable accuracy but underestimated variation in metabolic power across conditions.*

Equation 1: for clarity, please right-justify the equation number so it is clearly separate from the equation itself.

I adapted this in the manuscript

**Reviewer 2:**

1. The authors call the model a “neuro-musculoskeletal model”. Even though they do clearly describe what they mean with that, I still think that this term might put readers on the wrong foot. I agree that a neural model is included in the current work through the objective minimization, but it is not common to describe this as neuro-musculoskeletal model in the current literature. A neuro-musculoskeletal model usually implies that a neural control model is included, i.e., a set of mathematical equations describing the neural control as a function of the sensory inputs (e.g. a reflex model). Furthermore, because there is the musculoskeletal model and the objective, there are really two separate model types, and “neuro-musculoskeletal model” implies that it is a single thing.

We agree with the reviewer that this term might be misleading. The model is now called musculoskeletal model throughout the manuscript when we write about our specific simulation workflow.

2. Line 38, 216. The authors claim that the implemented tasks are straightforward, but there are some obvious differences between how the tasks were measured experimentally and how they were implemented. Most notable, there is a difference between treadmill walking and overground walking. Therefore, I would recommend wording changes throughout the manuscript and clarifying these differences throughout the manuscript.

2a. Line 38: “ensuring” seems a bit too strong here.

We adapted this in the manuscript, this line now reads:

*We chose tasks that are relatively straightforward to model to limit the contribution of errors in modeling the task to prediction errors.*

2b. Line 94: “ensuring that prediction errors …” is also too strong.

We adapted this in the manuscript, this line now reads:

*We specifically chose conditions that are relatively straightforward to model, to minimize the risk that prediction errors stem from shortcomings in the musculoskeletal and neural control models, rather than inadequate modeling of the intervention itself.*

2c. Line 216: a simulation with a forward pelvis speed is actually different from a treadmill experiment.

We agree with the reviewer’s remark. While overground walking and walking on a constant-speed treadmill are mechanically equivalent, treadmills typically do not operate at perfectly constant speeds. Additionally, subjects may walk slightly differently on a treadmill due to altered sensory inputs, such as visual cues (see adaptations in manuscript below questions 2d).

2d. The discussion should include more reflection on the difference between experiment and simulation. Would a treadmill model improve the accuracy of the simulations? Are there any other differences? The reflection could also include more reflection on the metabolic power over- and underestimation (line 362-364) and on why some of the experiments were implemented differently in the simulation than in the experiment (line 223 and 336).

We have added a short section to the Discussion to clarify that the walk-to-run transition was imposed in some experiments but was never imposed in the simulations. We also discuss the limitation of not modeling the treadmill and assuming a constant treadmill speed (see our response to the previous question).

New paragraph in discussion section:

*Although we selected tasks that were relatively easy to model, our simulations did not fully capture the experimental conditions. First, we used treadmill walking data to benchmark the simulations at various gait speeds but did not explicitly model walking on a treadmill. The treadmill belt speed is often not perfectly constant (e.g., belt speed is often lower than desired at heel strike) due to interactions between the human and treadmill and accounting for these interactions would require us to model the treadmill as well. In addition, walking on a treadmill alters sensory inputs (e.g. from vision) but our simulation framework does not capture sensorimotor control. Second, in the studies of Van der Zee et al.* [10] *and Abe et al.* [11]*, participants were required to walk at all speeds up to 2 m/s and to run at higher speeds, whereas the simulations were free to adopt either gait pattern at each speed (Figure S15). This discrepancy mainly affects comparisons around the walk-to-run transition (~2 m/s). Third, experiments were performed in a group of participants whereas simulates were performed based on a single musculoskeletal model with fixed anthropometry rather than a set of virtual participants with varying body parameters* [12]*. We chose this approach for computational reasons and because differences between simulations and average experimental data well exceeded inter-subject differences in experimental data after normalization. However, it is likely that the effect of errors in the musculoskeletal models, metabolic energy models, and cost function were more important than the errors in representing the task given the magnitude of differences between experimental and simulated metabolic power.*

3. Line 101: I would not say that the objective “typically comprises” all those terms listed in lines 100-102, based on the literature. Please rewrite. While the objective has been used in at least 2-3 papers from the authors’ group, there are many more predictive simulation studies that use other objectives, such as….

We thank the reviewer and have rephrased these lines; they now read:

*Physics-based simulations typically assume that humans select a gait pattern, from the redundant set of kinematic trajectories, that minimizes a cost function representing the cost to the human of achieving a task (1). The cost function typically compromises terms related to muscle fatigue (e.g. maximum muscle activations (5)), signal-dependent motor noise (e.g. activations squared (6)), musculoskeletal loading (e.g. contact forces (7)), movement smoothness (e.g. joint accelerations (8)), and metabolic power (9).*

Please note that we added some terms here that were not in the objective function we used such as maximal activations, contact forces, …

4. Relatedly, I think that the introduction and discussion could explore the current literature on predictive simulations more. The authors made specific (and logical) choices for the objective and modelling of the musculoskeletal model, but for both, there are other options. For the objective, it is as described above, while other studies in the list above have included body variations through virtual participants in different ways. It would be good to describe these options in more detail in the introduction and also reflect on those in the discussion. For example, I would expect that the results in, e.g., Fig 3, are highly dependent on the choice of walking objective.

We thank the reviewer for this suggestion. The first reviewer also recommended expanding the discussion of the objective function and its influence on our results. We have therefore added a substantial section to the Discussion addressing this point. We chose not to expand the Introduction in detail on these alternatives, in order to keep it focused and avoid confusion.

Regarding virtual participants, since the published experimental results we used are typically averages across subjects, we decided not to simulate a set of virtual participants with varying anthropometry. Instead, we non-dimensionalized the experimental outcomes and recomputed them for the anthropometry of our specific musculoskeletal model. We also discuss this in a limitation paragraph in the Discussion (see answer to your question 2 c/d for adaptations in manuscript).

5. Line 191: normally, the initial state of the simulation is also identified through the optimal control problem. Please add that to the description

It is important to note that, all (time-discretized) states including the initial states are optimized, under the constraint that all states are fully periodic (i.e., initial states equaled end states). Note that, technically speaking, the term initial state is a bit ill-defined here as we simulated a limit cycle.

6. Line 279: I am a bit confused by the description, because I interpreted the description as though the measured metabolic energy was used to calculate the efficiency and not the estimated net energy from the simulation? Or am I missing something?

I adapted this in the manuscript, this line now reads:

*The efficiency of net mechanical work done in the gait simulations was computed as the simulated net mechanical work by muscle fibers divided by the simulated net metabolic energy in one gait cycle. The net metabolic energy is the total metabolic energy minus the basal metabolic energy. The efficiency of positive mechanical work was computed as the simulated positive mechanical work done by muscles fibers divided by the simulated net metabolic energy. Mechanical work done by fibers is the time integral of muscle fiber power. Muscle fiber power was computed as a product of the velocity of the muscle fiber and its force. As the efficiency of net and positive mechanical work was unrealistically high compared to experiments* [13] *we also computed the maximal mechanical efficiency of muscle fibers in our model.*

7. Line 300: what is meant with, e.g., 10% overestimation and how was it calculated?

Using linear regression. I clarified this in the methods section, this line now reads:

*We quantified the relation between simulated and measured changes in stride frequency and gait cycle average metabolic power (referred to as metabolic power) across different walking conditions by computing the explained variance (*$R^{2})$*, root-mean square error (RMSE), and slope of the relation using a linear least squares fit. The slope was used to identify over or underestimation of metabolic power. For trajectory outcomes (joint kinematics and kinetics) we discussed the differences between experiments and simulations qualitatively.*

8. Results (e.g., Figure 2): In general, I am not sure if a simple correlation is valid for the comparisons that are made here. A simple correlation assumes independent data, but this is violated by the fact that there are multiple experiments. In fact, you can see an effect of the experiment in the graphs. I am not an expert myself, but I would think that something like a linear mixed model would be required to adjust for the experiment factor.

We thank the reviewer for this suggestion. While a linear mixed model could formally account for the experiment factor and might slightly adjust the slope between measured and simulated outcomes, we expect that it would not materially change the main conclusions of our study. We therefore opted to retain a simple correlation for clarity and transparency. All individual data points are plotted, and the full simulation methods and results are provided, allowing readers to explore alternative analyses, including mixed models, on any subset of the data if desired.

9. Figure 3: I did not fully understand the analysis that was done here and would need to dive back into the original experimental paper. Therefore, please explain figure D-G more, so that they can be interpreted without reading the original paper.

We thank the reviewer for the comment. We understand that Figures 3D–G are complex and that full interpretation requires familiarity with the original experimental paper. Unfortunately, the experimental protocol is too detailed to be fully reproduced in our manuscript, which is already lengthy (+/- 8000 words). Nevertheless, to help the reader, we have added brief clarifications in the figure caption and the Results section to highlight the key points necessary to interpret the figures without reading the original paper.

10. Figure 4: Please explain Figured C-F more. It is not clear how these results are generated, specifically the different lines and the “number of muscles”.

This was described in the methods section: see section Mechanical efficiency of the metabolic energy model. We have now also added a brief description to the caption of this figure (4).

*As the efficiency of net and positive mechanical work was unrealistically high compared to experiments (16) we also computed the maximal mechanical efficiency of muscle fibers in our model. We did this by computing muscle fiber power and metabolic power in isokinetic contractions at different fiber contraction velocities while the muscle fiber was maximally activated and when the muscle fiber was at the optimal length*

11. Figure 6: Figure C is not referred to in the manuscript. Consider removing or adding a reference.

I added a reference to figure 6C.

12. How is the COT with reduced dissipation larger than without? I compared to the previously mentioned value, 5.03 in line 543. Please clarify

We agree that this finding is counterintuitive. With the stiffer ground contact model, energy dissipation in the contact itself was reduced, but the positive and negative muscle fiber work increased, resulting in a higher overall cost of transport. We have added this explanation to the Discussion section for clarity.

*A less compliant ground contact model [14] reduced dissipation to 15 J/stride but led to a higher estimated COT of 6.46 J/kg/m with the Margaria energy model for level walking at 1.3 m/s due to increases in positive and negative fiber work.*

13. Line 600-601: the comparison that is made here is unfair. On the one hand, the simulation probably assumes an idealized exoskeleton with any possible control that spans all joints in the legs, while such an exoskeleton cannot be created in practice, and there are limitations in the connection between exoskeleton and person.

Yes, you are right and in fact this is exactly the point we want to make. We wrote: “*This demonstrates that other modelling assumptions might become critical in designs that have a large impact on the simulated walking motion and/or muscle activity*”. The modelling assumption here is a rigid contact between exoskeleton and human. We adapted the discussion section to make this stand out more. The line now reads:””.

*This demonstrates that other modelling assumptions (e.g. rigid contact between exoskeleton and human, influence on balance, …) might become critical in designs that have a large impact on the simulated walking motion and/or muscle activity.*

Minor comments

Thanks for all the minor comments.We corrected this in the manuscript. There is one comment we don’t agree with

14. Line 536: This paragraph only discusses inaccuracies in the muscle dynamics. Consider rephrasing.

We respectfully disagree. As noted, positive and negative muscle work is the result of musculoskeletal dynamics and not only of muscle dynamics. This the amount of positive and negative work done during a gait cycle is also influenced by the choice of the skeletal model and its model parameters, as well as its interaction with the environment and assumptions on, for example, dynamics of soft tissues.

**References**

[1]	M. Ackermann and A. J. van den Bogert, “Optimality principles for model-based prediction of human gait.,” *J. Biomech.*, vol. 43, no. 6, pp. 1055–60, Apr. 2010, doi: 10.1016/j.jbiomech.2009.12.012.

[2]	K. A. McDonald, J. P. Cusumano, A. Hieronymi, and J. Rubenson, “Humans trade off whole-body energy cost to avoid overburdening muscles while walking,” *Proc. R. Soc. B Biol. Sci.*, vol. 289, no. 1985, p. 20221189, doi: 10.1098/rspb.2022.1189.

[3]	L. J. Holmberg and A. Klarbring, “Muscle decomposition and recruitment criteria influence muscle force estimates,” *Multibody Syst. Dyn.*, vol. 28, no. 3, pp. 283–289, Sep. 2012, doi: 10.1007/s11044-011-9277-4.

[4]	K. Sasaki, R. R. Neptune, and S. A. Kautz, “The relationships between muscle, external, internal and joint mechanical work during normal walking,” *J. Exp. Biol.*, vol. 212, no. 5, pp. 738–744, Mar. 2009, doi: 10.1242/jeb.023267.

[5]	A. D. Koelewijn, D. Heinrich, and A. J. van den Bogert, “Metabolic cost calculations of gait using musculoskeletal energy models, a comparison study,” *PLOS ONE*, vol. 14, no. 9, p. e0222037, Sep. 2019, doi: 10.1371/journal.pone.0222037.

[6]	L. J. Bhargava, M. G. Pandy, and F. C. Anderson, “A phenomenological model for estimating metabolic energy consumption in muscle contraction $,” vol. 37, pp. 81–88, 2004, doi: 10.1016/S0021-9290(03)00239-2.

[7]	B. R. Umberger, “Stance and swing phase costs in human walking,” *J. R. Soc. Interface*, vol. 7, no. 50, pp. 1329–1340, 2010, doi: 10.1098/rsif.2010.0084.

[8]	B. R. Umberger, K. G. Gerritsen, and P. E. Martin, “A model of human muscle energy expenditure,” *Comput. Methods Biomech. Biomed. Engin.*, vol. 6, no. 2, pp. 99–111, 2003, doi: 10.1080/1025584031000091678.

[9]	K. Mombaur, A. Truong, and J.-P. Laumond, “From human to humanoid locomotion—an inverse optimal control approach,” *Auton. Robots*, vol. 28, no. 3, pp. 369–383, Apr. 2010, doi: 10.1007/s10514-009-9170-7.

[10]	T. J. van der Zee, E. M. Mundinger, and A. D. Kuo, “A biomechanics dataset of healthy human walking at various speeds, step lengths and step widths,” *Sci. Data*, vol. 9, no. 1, Art. no. 1, Nov. 2022, doi: 10.1038/s41597-022-01817-1.

[11]	D. Abe, Y. Fukuoka, and M. Horiuchi, “Economical Speed and Energetically Optimal Transition Speed Evaluated by Gross and Net Oxygen Cost of Transport at Different Gradients,” *PLOS ONE*, vol. 10, no. 9, p. e0138154, Sep. 2015, doi: 10.1371/journal.pone.0138154.

[12]	E. Dorschky *et al.*, “Optimal control simulation predicts effects of midsole materials on energy cost of running,” *Comput. Methods Biomech. Biomed. Engin.*, vol. 22, no. 8, pp. 869–879, Jun. 2019, doi: 10.1080/10255842.2019.1601179.

[13]	C. J. Barclay and N. A. Curtin, “Advances in understanding the energetics of muscle contraction,” *J. Biomech.*, vol. 156, p. 111669, Jul. 2023, doi: 10.1016/j.jbiomech.2023.111669.

[14]	L. D’Hondt, F. D. Groote, and M. Afschrift, “A dynamic foot model for predictive simulations of human gait reveals causal relations between foot structure and whole-body mechanics,” *PLOS Comput. Biol.*, vol. 20, no. 6, p. e1012219, Jun. 2024, doi: 10.1371/journal.pcbi.1012219.
