# Supplementary material for: Benchmarking the predictive capability of human gait simulations
Source: PLoS Comput Biol. 2025 Nov 17;21(11):e1012713. doi: 10.1371/journal.pcbi.1012713 (PMC12622833; doi:10.1371/journal.pcbi.1012713)
Supplement: Attachment — Submitted filename: Answer_reviewers.docx [file pcbi.1012713.s006.docx]

Dear Editor and reviewers,

Thank you again for taking the time to review our research article. We have carefully revised the manuscript based on your valuable feedback.

Reviewer 1:

We corrected several typing errors:

Line 96, "principals" is now "principles".

Line 769, "waking" is now "walking".

Reviewer 2:

We revised Line 611 in response to the reviewer’s comment that the phrasing “It is most likely a good idea” was too weak. The sentence now reads: **It is recommended to replace** activation terms by volume-scaled activation terms as the activation cost is sensitive to splitting muscles in different bundles whereas volume-scaled activation is not (56)

Regarding the reviewer’s main comment (paragraph starting at line 681): *Main comment (paragraph from line 681 onwards): I want to point the authors to an alternative explanation for the unrealistic kinematics and kinetics that could also be mentioned in addition: when using it as an objective, the optimization favors using lengthening work because of its high efficiency, which even makes some of the positive work free in a way. This to me is different to energy optimization as it happens by humans and by other metabolic models.*

We are afraid that we do not fully understand this comment and therefore cannot address it properly. As walking requires almost no net mechanical work (only to compensate for energy dissipation during ground contact), the sum of positive and negative muscle fiber work is approximately zero. Consequently, when minimizing the Margaria energy model, the optimization will minimize both positive and negative fiber work independently of their relative efficiencies. For this reason, we did not modify the manuscript based on this comment.
